# L-Carnitine Protects against Carboplatin-Mediated Renal Injury: AMPK- and PPARα-Dependent Inactivation of NFAT3

**DOI:** 10.1371/journal.pone.0104079

**Published:** 2014-08-04

**Authors:** Yuh-Mou Sue, Hsiu-Chu Chou, Chih-Cheng Chang, Nian-Jie Yang, Ying Chou, Shu-Hui Juan

**Affiliations:** 1 Department of Nephrology, Taipei Medical University-Wan Fang Hospital, Taipei, Taiwan; 2 Department of Anatomy, School of Medicine, College of Medicine, Taipei Medical University, Taipei, Taiwan; 3 Graduate Institute of Medical Sciences, Taipei Medical University, Taipei, Taiwan; 4 Department of Physiology, School of Medicine, College of Medicine, Taipei Medical University, Taipei, Taiwan; National Cancer Institute, United States of America

## Abstract

We have previously shown that carboplatin induces inflammation and apoptosis in renal tubular cells (RTCs) through the activation of the nuclear factor of activated T cells-3 (NFAT3) protein by reactive oxygen species (ROS), and that the ROS-mediated activation of NFAT3 is prevented by N-acetyl cysteine and heme oxygenase-1 treatment. In the current study, we investigated the underlying molecular mechanisms of the protective effect of L-carnitine on carboplatin-mediated renal injury. Balb/c mice and RTCs were used as model systems. Carboplatin-induced apoptosis in RTCs was examined using terminal-deoxynucleotidyl-transferase-mediated dUTP nick end labeling. We evaluated the effects of the overexpression of the peroxisome-proliferator-activated receptor alpha (PPARα) protein, the knockdown of PPARα gene, and the blockade of AMPK activation and PPARα to investigate the underlying mechanisms of the protective effect of L-carnitine on carboplatin-mediated renal injury. Carboplatin reduced the nuclear translocation, phosphorylation, and peroxisome proliferator responsive element transactivational activity of PPARα. These carboplatin-mediated effects were prevented by L-carnitine through a mechanism dependent on AMPK phosphorylation and subsequent PPARα activation. The activation of PPARα induced cyclooxygenase 2 (COX-2) and prostacyclin (*PGI2*) *synthase* expression that formed a positive feedback loop to further activate PPARα. The coimmunoprecipitation of the nuclear factor (NF) κB proteins increased following the induction of PPARα by L-carnitine, which reduced NFκB transactivational activity and cytokine expression. The in vivo study showed that the inactivation of AMPK suppressed the protective effect of L-carnitine in carboplatin-treated mice, indicating that AMPK phosphorylation is required for PPARα activation in the L-carnitine-mediated protection of RTC apoptosis caused by carboplatin. The results of our study provide molecular evidence that L-carnitine prevents carboplatin-mediated apoptosis through AMPK-mediated PPARα activation.

## Introduction

The quaternary ammonium compound, L-carnitine (L-trimethyl-3-hydroxy-ammoniabutanoate), is synthesized in cells from lysine and methionine precursors [1], and is required for the transport of fatty acids from the cytosol into the mitochondria during lipid catabolism. It has been sold as the nutritional supplement vitamin Bt, and has been used as a growth factor for mealworms. In cells, L-carnitine induces antioxidant proteins, including endothelial nitric oxide synthase, heme oxygenase-1 (HO-1), and super oxide dismutase (SOD) [2], and protects against lipid peroxidation in phospholipid membranes and oxidative stress in cardiomyocytes and endothelial cells [3]. In addition, L-carnitine protects renal tubular cells (RTCs) from gentamicin-induced apoptosis through prostaglandin (PG) I2-mediated activation of the peroxisome-proliferator-activated receptor (PPAR) α protein [4].

The second-generation platinum-containing anticancer drug, carboplatin (*cis*-diammine-1,1-cyclobutanedicarboxylate platinum II), is used to treat lung, ovarian, and head and neck cancers [5]. The antitumor action of carboplatin is mediated by the alkylation of DNA, which can lead to cell death in tumor cells. Carboplatin is more water-soluble and has fewer adverse effects than its analog, cisplatin, and has equivalent DNA-damaging activity as cisplatin at similar toxic doses [6]. Cisplatin is a potent chemotherapy agent used to treat various malignant cancers, but the doses are limited due to its detrimental effects in renal tubular function and a decline in the glomerular filtration rate [7], [8]. Because carboplatin has fewer toxic adverse effects than cisplatin, increased doses of carboplatin are commonly used in the clinic in order to achieve optimal antitumor effects. However, the predominant dose-limiting toxicities of carboplatin are bone marrow suppression and ototoxicity caused by free-radical oxidative injury [9]. Using both gain- and loss-of-function strategies, we previously showed that the activation of the transcription factor, nuclear factor of activated T cells-3 (NFAT3), induces RTC apoptosis, and that NFAT3-mediated apoptosis in RTCs is blocked by HO-1 gene therapy and N-acetyl cysteine (NAC) treatment [10]. The antioxidant activities of L-carnitine warrant further investigation to determine whether it might provide protection against carboplatin-mediated renal injury.

The ligand-activated transcription factors, PPARα and PPARγ, form a heterodimer with the retinoid X receptor, and bind to peroxisome proliferator responsive elements (PPREs) in target genes [11], [12]. The activities of PPARα and PPARγ are also regulated by phosphorylation [13], [14]. We have previously shown that the activation of PPARα by adenosine-monophosphate-activated protein kinase (AMPK) is dependent on the adiponectin-induced activation of HO-1 and cyclooxygenase (COX)-2 [15], [16]. In addition, PGI2 might be a ligand of PPARα and PPARδ [17], [18]. Garrelds et al. (1994) reported that PGI2 expression significantly increased in rat peritoneal leukocytes after a short-term (4 d) L-carnitine treatment [18]. Recent studies have also revealed that the L-carnitine-induced expression of PGI2 can induce the vasodilation of subcutaneous arteries in humans [19], [20]. Therefore, investigations of the mechanism by which the interwoven relations of PGI2 and PPARα are involved in protection of L-carnitine in carboplatin-challenged RTCs are warranted.

The activation of PPARα has been shown to play a beneficial role in preventing various diseases by inhibiting the NFκB-induced expression of inflammatory mediators, including vascular cell adhesion molecule-1, interleukin (IL)-6, endothelin-1, and tissue factor, in a broad range of cells, including endothelial cells, smooth muscle cells, and macrophages [21]–[23]. The activation of PPARα by fibrates inhibits the IL-1-induced secretion of IL-6 in human aortic smooth muscle cells [24]. By contrast, the aorta of PPARα-null mice undergoes an exacerbated response to lipopolysaccharide, demonstrating that the anti-inflammatory effect of fibrates on the vascular wall requires PPARα activation in vivo [25]. In addition, PPARα ligands also regulate hepatic inflammation, and fibrates reduce serum levels of acute-phase proteins, such as C-reactive protein (CRP) and fibrinogen [26]. Evidence from clinical trials also supports the role of PPARα ligands in suppressing inflammation. Fenofibrate treatment reduces the plasma concentrations of fibrinogen, IL-6, CRP, interferon-γ, and tumor necrosis factor (TNF)-α in patients with hyperlipidemia and atherosclerosis [24], [27]. Additionally, PGI2 and PPARα have been shown to protect against ischemia-reperfusion injury through the suppression of inflammation [28].

In our current study, we evaluated the protective effects of L-carnitine on carboplatin-mediated renal injury in vitro and in vivo. We also investigated the mechanisms underlying the PPARα-dependent suppression of carboplatin-mediated NFAT3 activation and inflammation.

## Materials and Methods

### Cell culture and reagents

We used the rat renal proximal tubular epithelial cell line, NRK-52E, for the in vitro *RTC model in our study*. NRK-52E epithelial cell lines are composed of differentiated, anchorage-dependent, nontumorigeic cells that undergo density-dependent inhibition of proliferation [29]. The widely used NRK-52E rat kidney cell lines have been characterized with the morphological and kinetic properties of kidney tubule epithelial cells [30]. The NRK-52E cells were purchased from the Bioresource Collection and Research Center (Hsinchu, Taiwan) and were cultured in Dulbecco’s modified Eagle medium (DMEM) supplemented with 10% fetal bovine serum (FBS) and an antibiotic and antifungal solution. The NRK-52E cell monolayers were grown until confluence was reached. The DMEM, FBS, and other tissue culture reagents were obtained from Life Technologies (Gaithersburg, MD, USA). The L-carnitine was purchased from Sigma-Tau (Rome, Italy). All of the other chemicals were of reagent grade, and were purchased from Sigma-Aldrich (St. Louis, MO, USA).

### Plasmid construction and expression analysis of the PPAR and the NFκB enhancers

A pBV-luc plasmid containing the prototypic sequence of the PPAR response element, 5′-AGGTCAAAGGTCA-3′, from the acyl-CoA oxidase gene promoter was provided by Dr. Vogelstein of Johns Hopkins University [31]. The NFκB-luciferase reporter plasmid, which contains the multimeric NFκB regulatory element, (TGGGGACTTTCCGC)_5_, was purchased from Stratagene (La Jolla, CA, USA). The RTC cells were transfected with these vectors using the LipofectAMINE 2000 (Invitrogen, Carlsbad, CA, USA) transfection reagent. After transfection for 4 h, the medium was replaced with complete medium, and the transfected cells were incubated for an additional 20 h. The transfected cells were treated with carboplatin for 2 h. The luciferase activity of the cell lysates were recorded using the Dual Luciferase Assay Kit (Promega, Madison, WI, USA) in a TD-20/20 luminometer (Turner Designs, Sunnyvale, CA, USA). The luciferase activity of the reported plasmids was normalized to that of the empty reporter plasmid and the pRL-TK Renilla luciferase plasmid.

### Small interfering RNA-mediated gene silencing of PPARα

The PPARα small interfering (si) RNA duplexes, 5′-GAACAUCGAGUGUCGAAUATT-3′ and 5′-GACUACCAGUACUUAGGAATT-3′ were purchased from Ambion (Austin, TX, USA). The RTCs were seeded in 6-well plates, and were transfected for 24 h using 100 pmol of the PPARα siRNAs, or the scrambled siRNAs in 100 µL of siPORTNeoFX. The expression of PPARα and other relevant proteins was analyzed using western blotting.

### Co-immunoprecipitation and western blot analysis of cytosolic and nuclear fractions of cell lysates

The PPARα protein was immunoprecipitated in samples containing 200 µg of total protein using 2 µg of an anti-PPARα antibody and 20 µg of protein-A-plus-G agarose beads to determine whether the p65 and/or p50 proteins coprecipitated with the PPARα protein. The precipitates were washed 5 times with a lysis buffer and once with phosphate-buffered saline (PBS). The washed pellet was resuspended in a sample buffer containing 50 mM Tris, 100 mM bromophenol blue, and 10% glycerol at pH 6.8, and incubated at 90°C for 10 min. The precipitated proteins were released from the agarose beads during gel electrophoresis. The RTCs were cultured in 10-cm^2^ dishes. The RTCs were pretreated using 5 mM L-carnitine for 24 h, and were harvested after carboplatin challenge for the indicated time points. The cell lysates were partitioned into cytosolic and nuclear fractions using the NE-PER nuclear extraction reagents (Pierce, Rockford, IL, USA) and protease inhibitors.

The western blotting procedure has been described elsewhere [15]. The following antibodies were used in the western blot analysis at the dilutions indicated: antibodies against the NFAT3, PTEN, PPARα, Bcl-xL, Bcl-xS, NFκB-p65, NFκB-p50, PGIS, lamin A/C (1∶1000; Santa Cruz Biotechnology, Dallas, TX, USA), pPPARα-Ser21 (1∶500; ABR Affinity Bioreagents, Rockford, IL, USA), cleaved caspase-3, COX-2 (1∶500; Cayman Chemical, Ann Arbor, MI, USA), GAPDH (1∶2000; Ab Frontier, Seoul, Korea), AMPK and phospho-AMPK proteins (1∶500; Millipore, Burlington, MA, USA). Aliquots of the nuclear and cytosolic fractions containing 50 µg of total protein were separated on a 10% acrylamide gel using sodium dodecyl sulfate-polyacrylamide gel electrophoresis. The protein bands in the acrylamide gel were electrophoretically transferred to a Hybond-P membrane (GE Healthcare Life Sciences, Waukesha, WI, USA), and the membranes were probed using the various primary antibodies. Band intensities in the western blots were normalized based on GAPDH (control) band intensity using an IS-1000 digital imaging system (ARRB, Victoria, Australia).

### Analysis of gene expression using a reverse-transcription polymerase chain reaction

A previously described method was used to obtain the total RNA for the analysis of gene expression using a reverse-transcription polymerase chain reaction (RT-PCR), with minor modifications (Pang et al., 2008). Sequences of the primer pairs used for the amplification of each gene were as follows: 5′-TGCCTCAGCCTCTTCTCATT-3′ and 5′-CCCATTTGGGAACTTCTCCT-3′ for the TNFα gene (108 bp); 5′-AGGTATCCATCCATCCCACA-3′ and 5′-GCCACAGTTCTCAAAGCACA-3′ for the ICAM-1 gene (209 bp); 5′-ATGCAGTTAATGCCCCACTC-3′ and 5′-TTCCTTATTGGGGTCAGCAC-3′ for the MCP-1 gene (167 bp); and 5′-AACTTTGGCATTGTGGAAGG-3′ and 5′-TGTTCCTACCCCCAATGTGT-3′ for the GAPDH gene (223 bp). In each experiment, 5 µg of total RNA from the extracts of RTCs was used. The total cDNA in each RT-PCR sample was normalized to that of the GAPDH samples. The PCR products were separated on a 2% agarose gel and quantified using an electrophoresis image analysis system (Eastman Kodak, Rochester, NY, USA).

### Animals and treatments

All animal study procedures were conducted in accordance with the Taipei medical university animal care and use rules (licenses No. LAC-101-0102) and an Association for Assessment and Accreditation of Laboratory Animal Care approved protocol. Eight-week-old male Balb/c mice weighing 20 to 25 g were obtained from the Research Animal Center at National Taiwan University (Taipei, Taiwan). The animals were housed in a central facility, were subjected to a 12-h light–dark cycle, and were given regular rat chow and tap water. The mice were separated into the control, carboplatin, carboplatin+L-carnitine, carboplatin+L-carnitine+compound C (an AMPK inhibitor), compound C groups, carboplatin+compound C, and L-carnitine, with 12 mice in each group except for the compound C group with 16 mice. Compound C (10 mg/kg) was intraperitoneally injected 1 h before the L-carnitine was administered. The L-carnitine (50 mg/kg) or compound C was given 2 days before a single dose of carboplatin (75 mg/kg) was intraperitoneally injected. Within the 4-day period of carboplatin challenge, L-carnitine and compound C were given every 2 days.

At the end of the treatment period, animals were anaesthetized intramuscularly with a combination of ketamine (8 mg/100 g body weight), xylazine (2 mg/100 g) and atropine (0.16 mg/100 g). Mice’s blood samples were collected to measure the serum levels of creatinine and urea nitrogen using Fuji Dri-Chem slides (Fujifilm, Tokyo, Japan). The kidneys were harvested by performing a laparotomy, and tissue samples of the renal cortex were snap-frozen in dry ice before being stored at −80°C. The kidney tissue samples were fixed in 10% formalin, and embedded in paraffin. Serial 5-µm sections were prepared from the paraffin-embedded samples from the control and carboplatin-treated groups, and the sections were stained with hematoxylin and eosin (H&E) for histological analysis that was performed by a pathologist in a single-blind fashion. Frozen sections (5-µm) were also prepared for terminal deoxynucleotidyl transferase dUTP nick end labeling (TUNEL) 4 d after carboplatin treatment.

### Statistical analysis

The data are expressed as the mean ± the standard deviation (SD), and represent the results of at least 3 experiments. The means of the experimental and control groups were compared using a one-way analysis of variance, or the Bonferroni method was used for the post-hoc analysis. A value of *P*<0.05 was considered to indicate a statistically significant difference.

## Results

### Protection of L-carnitine in carboplatin-mediated inflammation and apoptosis in RTCs

**Figure 1:**
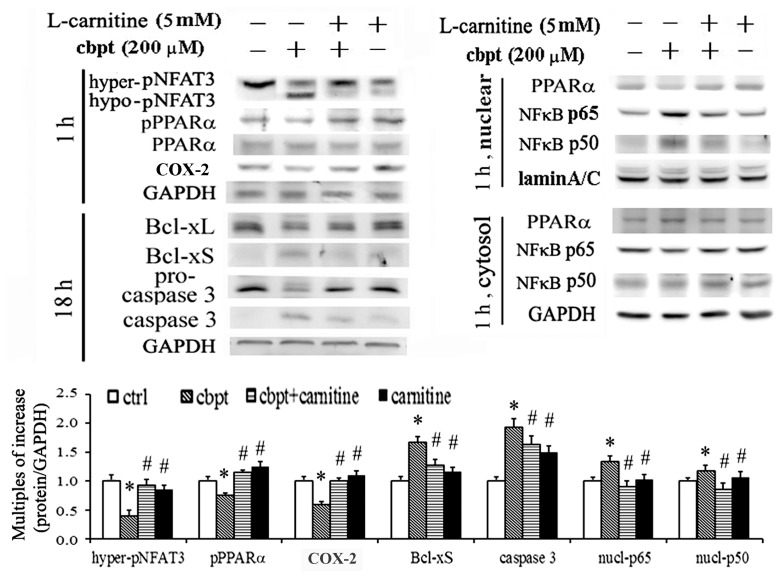
The protective effect of L-carnitine on the cytotoxicity of carboplatin in RTCs. The RTCs were pretreated with L-carnitine (5 mM) for 24 h, followed by treatment using 200 µM carboplatin for 1 h or 18 h, and the levels of PPARα, COX-2, Bcl-xL, Bcl-xS, and cleaved caspase-3 in the cell lysates were examined using a western blot analysis. The cell lysates of the samples with 1 h of carboplatin challenge were partitioned into cytosolic and nuclear factions. Band intensities were normalized based on GAPDH band intensity using densitometry. The bar chart shows the normalized intensities of each protein band. Lamin A/C and GAPDH were used as internal controls for the nuclear fraction and whole-cell lysate, respectively. Comparisons were subjected to ANOVA followed by Bonferoni’s post-hoc tests. Results are expressed as the mean ± SD (*n = *4). Data from a representative experiment are shown. Significant difference (*P<0.05 vs. the control; ^#^ *P*<0.05 vs. the Cbpt-treated group).

Because L-carnitine has antioxidant and antiapoptotic properties, the molecular mechanism of the protective effect of L-carnitine in carboplatin-challenged RTCs was examined using a western blot analysis. Our preliminary data showed that treatments using 5–40 mM L-carnitine were effective in prevention of carboplatin-mediated apoptosis in RTCs. Therefore, we used a low dose of 5 mM L-carnitine in our in vitro experiments. Carboplatin increased the levels of proteins involved in apoptotic and inflammatory signaling in RTCs, including hypo-pNFAT3, Bcl-xS, caspase 3, and p65/p50, and the levels of these proteins were significantly reduced in L-carnitine-treated cells. We observed that the protective effect of L-carnitine coincided with increased levels of PPARα and COX-2, whereas the levels of PPARα and COX-2 were reduced in cells treated using carboplatin alone (Figures 1).

### Signaling pathway of PPARα phosphorylation by L-carnitine in rescuing carboplatin-mediated changes in NFAT3 and PPARα activation in RTCs

**Figure 2:**
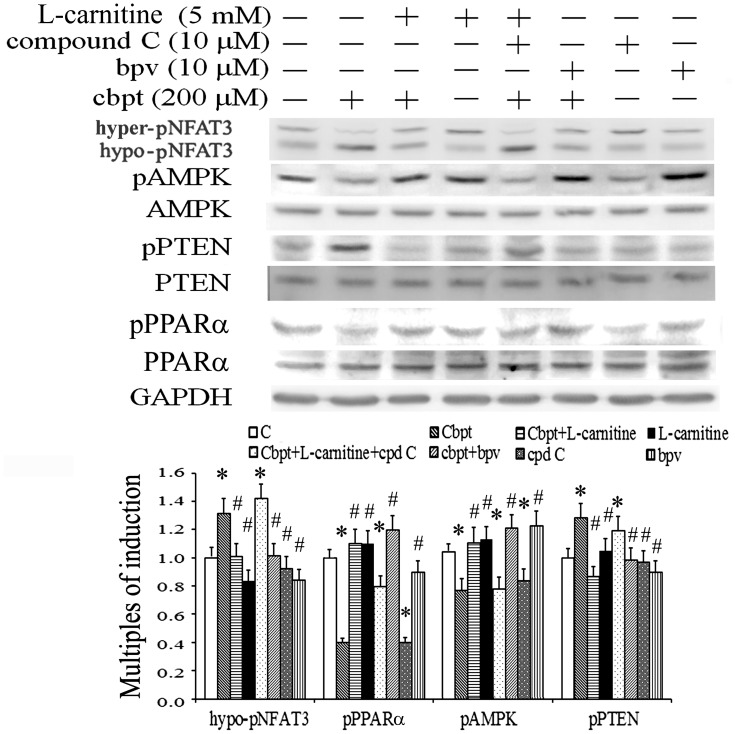
The activation of PPARα by L-carnitine in carboplatin-induced RTC injury. RTCs were pretreated with L-carnitine for 24 h or with the PTEN inhibitor BPV or the AMPK inhibitor compound C for 1 h, followed by carboplatin challenge for 20 min. The levels of NFAT3, AMPK, pPTEN, and pPPARα in the cell lysates were analyzed by conducting western blotting, using GAPDH as an internal control. The data represent the mean ± SD of the results of 3 independent experiments (*P<0.05 vs. the control; ^#^ *P*<0.05 vs. the Cbpt-treated group).

We have previously shown that NFAT3 is activated by ROS in carboplatin-challenged RTCs. We investigated the signaling pathways involved in the L-carnitine-induced NFAT3 inactivation but PPARα activation in RTCs treated with carboplatin by using a western blot analysis. As shown in Figure 2, carboplatin caused NFAT3 activation (hypo-phosphorylation) and PPARα inactivation (de-phosphorylation), which are correlated with the opposite phosphorylation status of PTEN and AMPK; increased phosphorylation of PTEN but reduced that of AMPK, compared with the phosphorylation of these proteins in the control cells. Nevertheless, L-carnitine reversed the activation of NFAT3 and PPARα caused by carboplatin through altering the phosphorylation of PTEN and AMPK.

The involvement of AMPK and PTEN by L-carnitine in the prevention of the dephosphorylation of NFAT3 and PPARα caused by carboplatin was verified using the AMPK inhibitor, compound C, and the PTEN inhibitor, BPV. Compound C inactivated AMPK, resulting in the elimination of L-carnitine-mediated PPARα phosphorylation in carboplatin-treated RTCs. By contrast, BPV inactivated PTEN, which coincided with increased AMPK and PPARα phosphorylation in carboplatin-treated RTCs. This suggests that L-carnitine activates PPARα through an AMPK/PTEN-dependent mechanism, resulting in the inactivation of NFAT3, which might be beneficial for the protection against carboplatin-mediated renal injury.

### PPARα overexpression mimics the protective effect of L-carnitine in carboplatin-mediated apoptosis and oxidative insult

**Figure 3:**
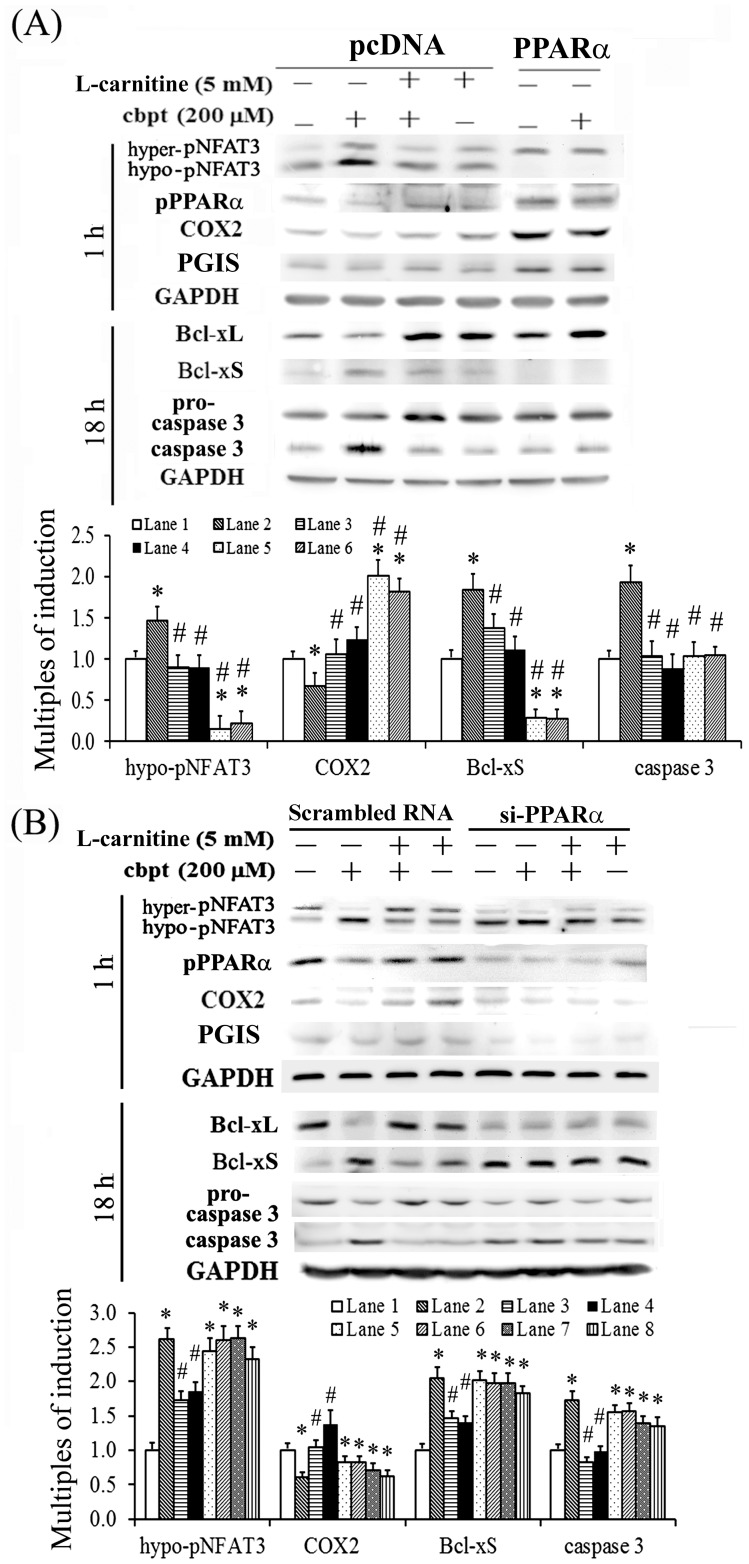
Overexpression of PPARα mimics the protective effect of L-carnitine in carboplatin-induced RTC apoptosis. Cells with PPARα overexpression (A) or PPARα silence (B) were pretreated with 5 mM L-carnitine for 24 h, followed by a 200-µM carboplatin challenge for 1 h and 18 h, and the levels of NFAT3, PPARα, Bcl-xL, Bcl-xS, and cleaved caspase-3 were analyzed by western blotting. The bar chart in each panel shows the normalized intensities of each protein band with GAPDH band using densitometry. Data were derived from the results of 4 independent experiments, and are presented as the mean ± SD (*P<0.05 vs. the control; ^#^ *P*<0.05 vs. the Cbpt-treated group).

We intend to unravel the causal relationship between PGI2 and PPARα signaling and their influence in the L-carnitine-mediated protection of carboplatin-treated RTCs. The essential role of PPARα in the antiapoptotic effect of L-carnitine in carboplatin-challenged RTCs was examined using both gain- and loss-of-function experimental designs. As shown in Figure 3A, the effect of PPARα overexpression mimicked the protective effect of L-carnitine with regard to the reduced ratio of Bcl-xS to Bcl-xL. Similar to the adverse effect of carboplatin, PPARα knockdown increased the ratio of Bcl-xS to Bcl-xL (Figure 3B), indicating the critical role of PPARα activation in the protective effect of L-carnitine. In addition, PPARα overexpression increased COX-2/PGIS expression in RTCs, whereas PPARα knockdown reduced COX-2/PGIS expression.

**Figure 4:**
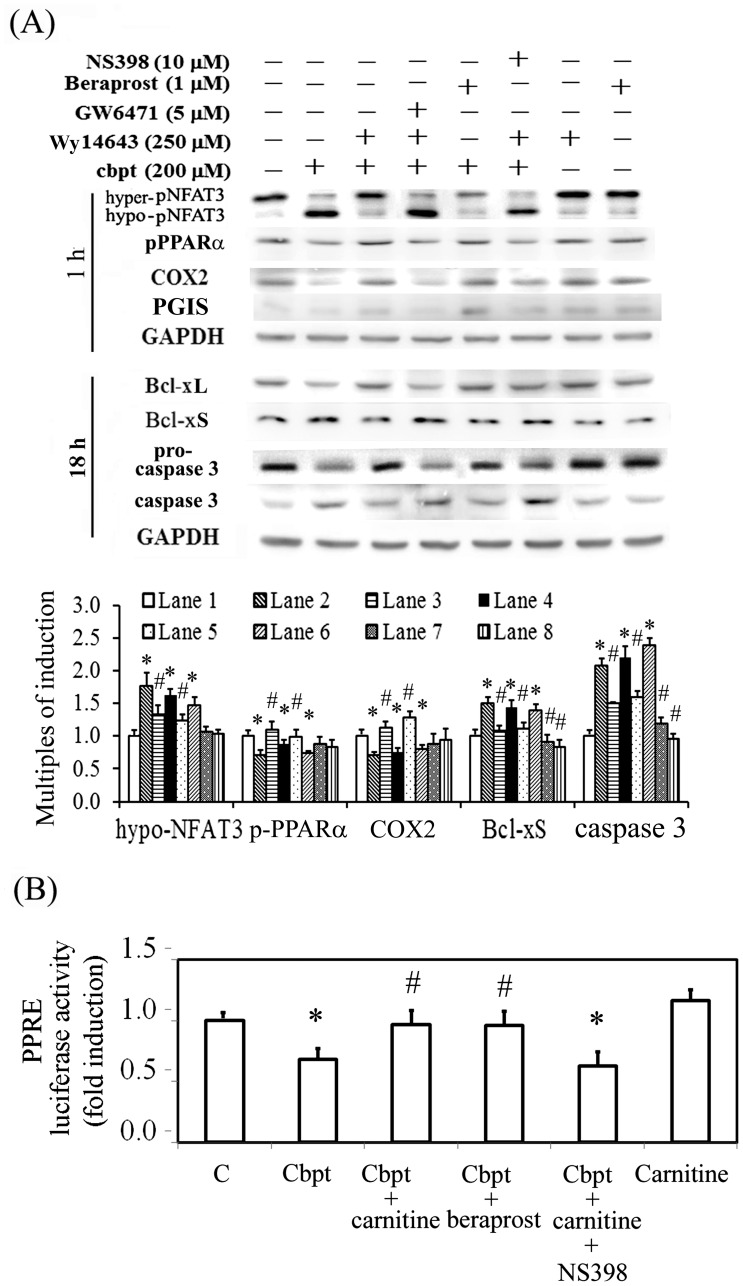
Causal relationship of PPARα, COX-2, and PGIS for the protective effect of L-carnitine in carboplatin-induced RTC apoptosis. (A) RTCs were pretreated using various antagonists/inhibitors and agonists of PPARα, COX-2, and/or PGI2 for 1 h or 5 mM L-carnitine for 24 h, followed by a 200-µM carboplatin challenge for 1 h and 18 h, and the levels of NFAT3, PPARα, Bcl-xL, Bcl-xS, and cleaved caspase-3 were analyzed by western blotting. Band intensities were normalized based on GAPDH band intensity using densitometry. The bar chart in each panel shows the normalized intensities of each protein band. (B) The RTCs were transfected with the PPRE-driven reporter plasmids overnight, and pretreated with 5 mM L-carnitine for 24 h, followed by a 200-µM carboplatin challenge for 2 h. The reporter luciferase activity was calculated by normalizing the intrinsic activity of samples based on the intensity of the pGL3-promoter vector and the transfection efficiency. Data were derived from the results of 4 independent experiments, and are presented as the mean ± SD (*P<0.05 vs. the control; ^#^ *P*<0.05 vs. the Cbpt-treated group).

We also examine the causal relationship among PPARα, COX-2, and PGIS in the L-carnitine-mediated protection of carboplatin-induced RTC apoptosis by assessing the effects of the PPARα agonist Wy14643, the PPARα antagonist GW6471, the PGI2 analog beraprost, and the selective COX-2 inhibitor NS398. As shown in Figure 4A, Wy14643 and beraprost mimicked the effect of L-carnitine in reducing the activation of NFAT3 and caspase 3, whereas GW6471 and NS-398 produced the opposite effect. In addition, Wy14643 induced COX-2 expression, which is consistent with the effects of PPARα overexpression and PPARα knockdown (Figure 3A and B). A PPRE-driven luciferase assay was used to examine whether the inhibitor and/or the analog of COX-2/PGIS affected the transactivational activity of PPAR. As shown in Figure 4B, carboplatin reduced the transactivational activity of PPAR. This reduction in PPARα transactivational activity was significantly reversed by treatment using additional L-carnitine or beraprost, whereas NS398 reduced the effect of L-carnitine in carboplatin-challenged RTCs. These data suggest that COX-2 and PGI2 (a feedback loop) are involved in the L-carnitine-induced increase in PPARα transactivational activity.

### Transactivational activity of NFκB is regulated by L-carnitine through PPARα binding of p65 and p50

**Figure 5:**
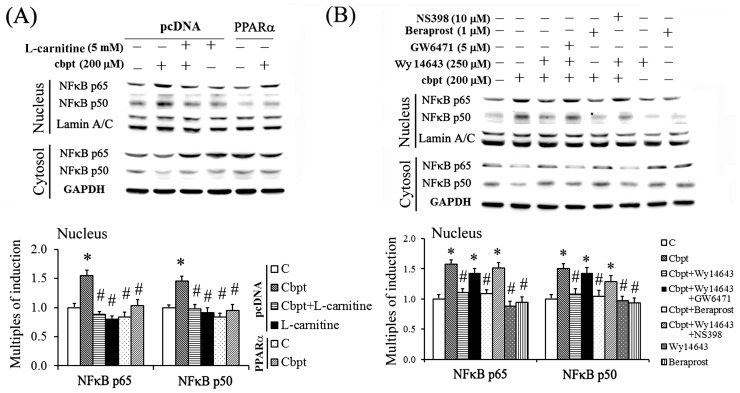
The inhibition of the carboplatin-mediated nuclear translocation of p65 and p50 by PPARα activation. The RTCs were (A) transfected with PPARα overnight and (B) pretreated using various antagonists/inhibitors and agonists of PPARα, COX-2, and/or PGI2 for 1 h, followed by carboplatin challenge for 1 h. The cell lysates were fractionated, and the cytosolic and nuclear distributions of p65 and p50 were analyzed using western blotting. Band intensities were normalized based on the GAPDH band intensity using densitometry. The bar chart in each panel shows the normalized intensities of each protein band. The data were derived from the results of 3 independent experiments, and are presented as the mean ± SD (*P<0.05 vs. the control; ^#^ *P*<0.05 vs. the Cbpt-treated group).

**Figure 6:**
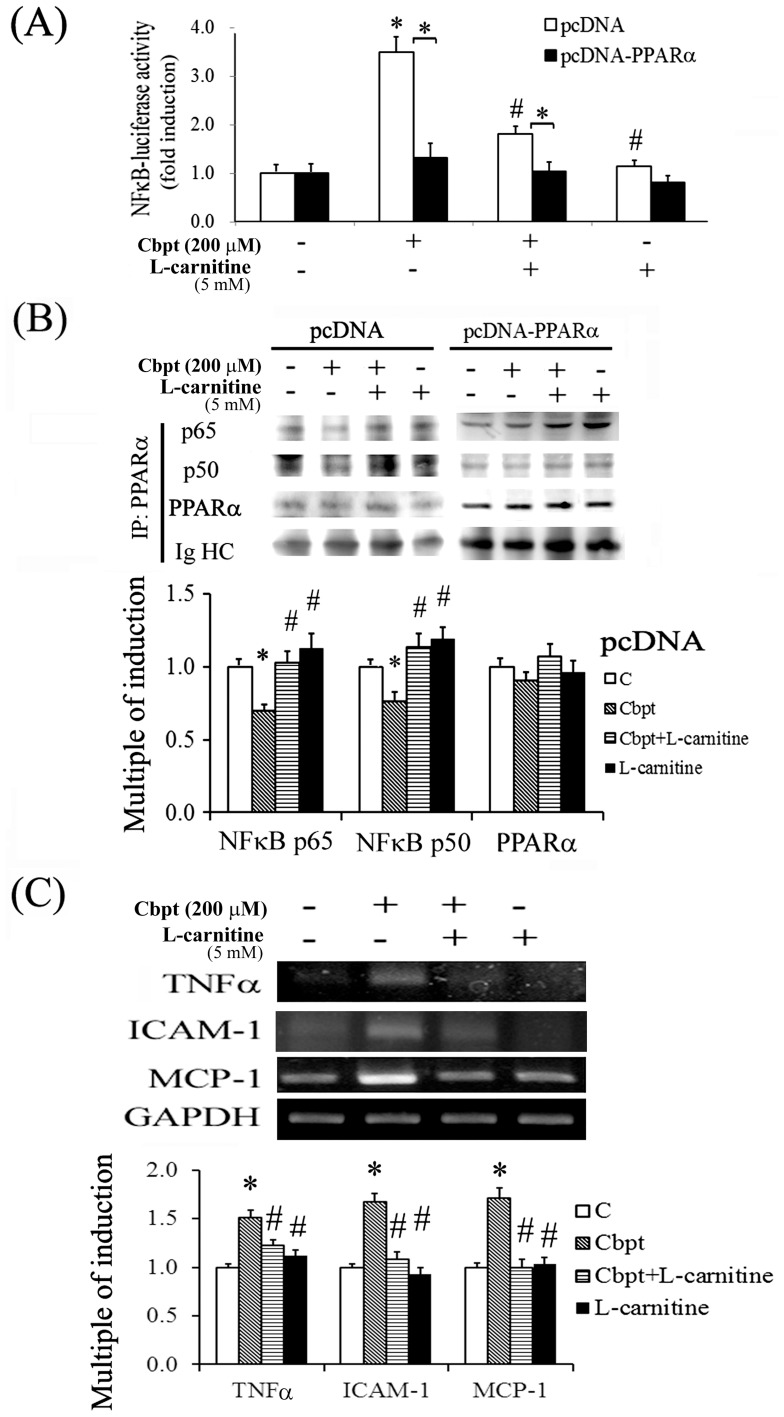
The inhibition of the carboplatin-mediated increase in NFκB activation by PPARα overexpression. (A) The RTCs were treated as described in Figure 3D, except the cells were transfected with the NFκB-luciferase vector. Luciferase activity was measured, and the data were processed as described in the Methods section. The data were derived from 3 independent experiments, and are presented as the mean ± SD (*P<0.05 vs. the control). (B) Cells were transfected with pcDNA or pcDNA-PPARα overnight, followed by L-carnitine or carboplatin treatment. The PPARα protein was immunoprecipitated using an anti-PPARα antibody, and the precipitates were probed using anti-p65 and anti-p50 antibodies. The data are representative of the results of 3 independent experiments. (C) The L-carnitine-mediated downregulation of inflammatory cytokines was analyzed using RT-PCR. The RTCs were pretreated using L-carnitine for 24 h, followed by carboplatin challenge for 4 h, to investigate the effect of L-carnitine on the carboplatin-mediated production of TNFα, MCP1, and ICAM1. The expression of GAPDH was used as an internal control. Comparisons were subjected to ANOVA followed by Bonferoni’s post-hoc tests. The data are presented as the mean ± SD of the results of 4 independent experiments (*P<0.05 vs. the control).

The mechanism underlying the anti-inflammatory effect of L-carnitine in carboplatin-treated RTCs was examined using genetic and pharmacological approaches. As shown in Figure 5, PPARα overexpression and treatments using agonists of PPARα and PGI2 reduced the carboplatin-mediated nuclear translocation of the NFκB-p65 and NFκB-p50 protein complexes, whereas the PPARα antagonist and COX-2 inhibitor eliminated the protective effect of L-carnitine in carboplatin-treated RTCs. The results of the NFκB-driven luciferase assay (Figure 6A) showed that carboplatin increased NFκB transactivational activity, and that treatment with additional L-carnitine or the overexpression of PPARα reversed the carboplatin-mediated increase in NFκB-transactivational activity in RTCs.

The mechanism underlying the PPARα-mediated reduction in NFκB activation was examined using immunoprecipitation. As shown in Figure 6B, both PPARα overexpression and L-carnitine-induced PPARα activation increased the amount of p65 and p50 that coprecipitated with PPARα, and reduced the transactivation activity of NFκB (Figures 5A and 6A). We examined the expression of ICAM, MCP-1, and TNFα in RTCs treated with L-carnitine and carboplatin to investigate the relationship between the levels of these cytokines and the regulation of NFκB transcriptional activity. The results of the RT-PCR assays showed that the L-carnitine treatment significantly reduced the expression of these inflammatory cytokines in carboplatin-treated RTCs relative to that in RTCs treated with carboplatin alone (Figure 6C).

### Essential roles of AMPK and PPARα activation in the L-carnitine-mediated protection of carboplatin-mediated renal injury in mice

**Figure 7:**
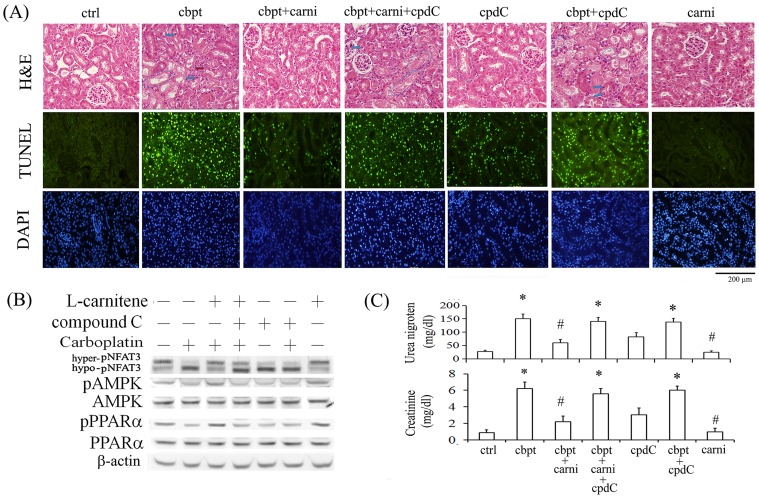
The inhibition of the protective effect of L-carnitine on carboplatin-mediated changes in renal structure and function in mice treated with an AMPK inhibitor. The kidneys were dissected and sectioned for (A) histological examination and TUNEL assay. Representative photographs of H&E staining are shown on top panel. Blue arrow: severe condense nuclei, red arrow: vacuolization of some proximal epithelial cells and blue circle: drop out of some epithelial cells. Apoptotic cells in the kidneys of experimental animals were detected in vivo using TUNEL staining. (Second panel) The TUNEL-labeled nuclei were visible as bright spots in the cortical sections of untreated and treated mouse kidneys. (Third panel) The identical fields were stained using DAPI to confirm the positions of the TUNEL-labeled cell nuclei. (B) The levels of NFAT3, AMPK and PPARα in the kidney extracts after mice with various treatments were evaluated using a western blot analysis using β-actin as an internal control. (C) The serum levels of urea nitrogen and creatinine in the 7 groups were measured at 4 days after carboplatin challenge. Comparisons were subjected to ANOVA followed by Bonferoni’s post-hoc tests. The results are expressed as the mean ± SD (*n* = 10; *P<0.05 vs. the control; ^#^ *P*<0.05 vs. the Cbpt-treated group).

The protective effect of L-carnitine in vitro was verified in carboplatin-challenged Balb/c mice, and the essential role of AMPK/PPARα activation in exerting protective effect of L-carnitine in carboplatin-induced renal injury was examined by using compound C, an AMPK inhibitor. As shown in Figure 7A, mice challenged with carboplatin showed severe condense nuclei (blue arrow), drop out of some epithelial cells (blue circle) and vacuolization of some proximal epithelial cells (red arrow). By contrast, the carboplatin-induced histological damage was milder in the mice treated with L-carnitine. Additional compound C treatment suppressed the protective effects of L-carnitine with regard to RTC structural integrity and histological changes with decreased number of epithelial cell nuclei in mice treated with carboplatin. Additionally, cell apoptosis in the kidney sections was evaluated by TUNEL assay with nuclei of cells stained with DAPI. The brightly stained nuclei produced by TUNEL were detected in the renal cortex of carboplatin-treated mice, but occurred rarely in those of the control and L-carnitine-treated mice. Most of the TUNEL-labeled nuclei were concentrated in proximal RTCs. These results demonstrate that carboplatin-induced apoptosis in mouse RTCs is inhibited by L-carnitine by approximately 75%. Additional compound C treatment reverted the protective effect of L-carnitine, increasing RTC apoptosis by approximately 65% compared with that of mice treated with both L-carnitine and carboplatin. The western blot analysis in Figure 7B showed that carnitine-mediated AMPK and PPARα activation in renal tissues was reverted in mice following compound C treatment, suggesting that L-carnitine exerts its protective effect on carboplatin-mediated renal injury through a mechanism that is dependent on the activation of AMPK and PPARα. Furthermore, in the renal functional assessment shown in Figure 7C, mice subjected to carboplatin insult showed increased serum levels of urea and creatinine, which suggests renal dysfunction. This can be significantly alleviated in mice additionally treated with L-carnitine, suggesting a marked prevention of renal function associated with carboplatin toxicity. However, levels of serum urea and creatinine in mice additionally treated with compound C were significantly higher than those observed in the carboplatin-L-carnitine group. These results suggest that L-carnitine protects against carboplatin-mediated renal injury through an AMPK/PPARα-dependent pathway, in which AMPK-dependent PPARα activation mediates the protective effect of L-carnitine.

## Discussion

We have previously shown that oxidative stress induces NFAT3 activation (hypophosphorylation) in carboplatin chemotherapy, resulting in inflammation and apoptosis in RTCs, and that carboplatin-induced NFAT3 activation is reduced by NAC treatment and HO-1 overexpression [10], [32]. The results of our current study provide both in vitro and in vivo evidence that L-carnitine inhibits carboplatin-mediated renal injury by suppressing the activation of NFAT3 and NFκB through AMPK-mediated PPARα activation, and that PPARα activation is essential to the protective effect of L-carnitine in RTCs.

Both L-carnitine and propionyl-L-carnitine (PLC), a carnitine derivative, induce endothelium-dependent vasodilation, and have been used for the treatment of cardiovascular diseases [33], [34]. Nitric oxide production in the aorta of hypertensive rats is enhanced by L-carnitine through PI3 and Akt kinases [35], and PLC promotes prostaglandin synthesis in subcutaneous arteries in humans [20]. We showed that the antioxidant and anti-inflammatory properties of L-carnitine protected RTCs from carboplatin-mediated injury. Although L-carnitine has been shown to induce SOD, catalase, and glutathione peroxidase [2], the antioxidant properties of L-carnitine were not addressed in our study.

Although Chen et al. [4] demonstrated that L-carnitine protects against gentamicin-mediated renal injury though a PGI2-PPARα pathway, we showed that PPARα regulated COX-2 and PGIS expression, which also increased PPARα transactivational activity, and the roles of PGI2, COX-2 and PGIS were confirmed in both gain- and loss-of-function experiments (Figure 3). This is in agreement with our previous finding that adiponectin-mediated COX-2 induction through a PPARα-dependent mechanism, and COX-2 exerted an anti-inflammatory effect of adiponectin in hepatocytes subjected to iron challenge [15]. In addition, a PGI2 agonist (beraprost) enhanced PPRE-driven transactivational activity (Figure 4B) and PPARα nuclear translocation, suggesting the existence of a positive feedback mechanism in the regulation of PPARα activation that has not been previously reported. Likewise, beraprost caused COX-2 upregulation (Figure 4A) through the positive loop of PGI2 in PPARα activation. Different to the concept of COX-2 as a pro-inflammatory molecule, we demonstrated that COX-2-mediated PGIS activation can directly or indirectly potentiate the PPARα activation, resulting in the anti-inflammation by suppressing NFκB activation in carboplatin-challenged RTC treated with L-carnitine. This finding is agreed by the study that COX-2 might induce anti-inflammatory effect by generating an alternative set of prostaglandins [36]. Additionally, nuclear factor erythroid-2-related factor-2 (Nrf2) has been proved to confer protection against oxidative stress [37]. Furthermore, COX-2-dependnet electophile oxo-derivative molecules have been shown to modulate the anti-inflammatory action via activation of Nrf2-dependent antioxidant response element (ARE) [38]. We also showed that AMPK regulates PPARα phosphorylation in L-carnitine-mediated protection in RTCs challenged with carboplatin (Figure 2), which is consistent with our previous finding that adiponectin protects hepatocytes from iron-overload-mediated apoptosis and inflammation through the AMPK-PPARα-mediated inductions of HO-1 and COX-2 [15], [16].

To gain insight into the mechanism by which PPARα is activated by L-carnitine, we focused our investigation on the relationship between AMPK and PTEN in PPARα activation. We demonstrated that a reciprocal relationship exists between AMPK and PTEN in the L-carnitine-mediated activation of PPARα in carboplatin-treated RTCs. These findings are consistent with those of a previous study, which showed that metformin, an insulin-sensitizing drug, suppressed the expression of PTEN through an AMPK-dependent mechanism in preadipocyte 3T3-L1 cells [39]. By contrast, AMPK knockdown eliminated the effects of metformin on reducing PTEN, suggesting that AMPK is involved in the regulation of PTEN. Additionally, we observed that L-carnitine stimulated the phosphorylation of PPARα through an AMPK/PTEN-dependent pathway. Although a previous study suggested that the phosphorylation of PTEN at S370 and S380 inhibits certain PTEN functions, how these phosphorylation events affect enzymatic activity is unclear [40]. We observed that carboplatin increased PTEN phosphorylation at S380, and that the carboplatin-mediated phosphorylation of PTEN is inhibited by the PTEN inhibitor BPV, which rescued the carboplatin-mediated reduction of AMPK phosphorylation. Thus, how PTEN phosphorylation affects PTEN enzymatic activity largely remains unclear. Future investigations are warranted to unravel the intricate relationship between AMPK and PTEN in L-carnitine-mediated PPARα activation.

The results of this study also demonstrated that L-carnitine reduces NFκB transactivational activity and then the production of TNFα, ICAM1, and MCP-1 in carboplatin-treated RTCs. A previous study showed that PPARγ modulates NFκB activity by interacting with the RelA/p65 subunit of NFκB in cells treated using TNFα, and this interaction was disrupted by exposure to cigarette smoke [41]. Likewise, in our immunoprecipitation experiments, we observed that PPARα directly interacted with NFκB (Figure 6B), and that PPARα-overexpression reduced NFκB transactivational activity in carboplatin-treated RTCs (Figure 6A). Additionally, both genetic and pharmacological activation of PPARα confirmed its involvement in the modulation of NFκB activation and NFκB-related inflammation through its effect on the nuclear translocation of p65 and p50 (Figure 5). Our findings are consistent with those of a previous study that showed that fibrates inhibit vascular inflammatory response through a PPARα-dependent reduction in NFκB and AP-1 transactivation [22]. Collectively, our results show that L-carnitine, a naturally occurring compound, can prevent the renal toxicity induced by carboplatin chemotherapy. The AMPK-induced activation of PPARα is essential to the protective effect of L-carnitine in carboplatin-mediated renal injury.
